# Both Paraoxonase-1 Genotype and Activity Do Not Predict the Risk of Future Coronary Artery Disease; the EPIC-Norfolk Prospective Population Study

**DOI:** 10.1371/journal.pone.0006809

**Published:** 2009-08-27

**Authors:** Rakesh S. Birjmohun, Menno Vergeer, Erik S. G. Stroes, Manjinder S. Sandhu, Sally L. Ricketts, Michael W. Tanck, Nicholas J. Wareham, J. Wouter Jukema, John J. P. Kastelein, Kay-Tee Khaw, S. Matthijs Boekholdt

**Affiliations:** 1 Department of Vascular Medicine, Academic Medical Center, Amsterdam, The Netherlands; 2 Department of Clinical Epidemiology and Biostatistics, Academic Medical Center, Amsterdam, The Netherlands; 3 Department of Cardiology, Academic Medical Center, Amsterdam, The Netherlands; 4 Medical Research Council Epidemiology Unit, Cambridge, United Kingdom; 5 Department of Cardiology, Leiden University Medical Center, Leiden, The Netherlands; 6 Department of Public Health and Primary Care, Institute of Public Health, University of Cambridge, Cambridge, United Kingdom; Innsbruck Medical University, Austria

## Abstract

**Background:**

Paraoxonase-1 (PON1) is an antioxidant enzyme, that resides on high-density lipoprotein (HDL). PON1-activity, is heavily influenced by the PON1-Q192R polymorphism. PON1 is considered to protect against atherosclerosis, but it is unclear whether this relation is independent of its carrier, HDL. In order to evaluate the atheroprotective potential of PON1, we assessed the relationships among PON1-genotype, PON1-activity and risk of future coronary artery disease (CAD), in a large prospective case-control study.

**Methodology/Principal Findings:**

Cases (n = 1138) were apparently healthy men and women aged 45–79 years who developed fatal or nonfatal CAD during a mean follow-up of 6 years. Controls (n = 2237) were matched by age, sex and enrollment time. PON1-activity was similar in cases and controls (60.7±45.3 versus 62.6±45.8 U/L, p = 0.3) and correlated with HDL-cholesterol levels (r = 0.16, p<0.0001). The PON1-Q192R polymorphism had a profound impact on PON1-activity, but did not predict CAD risk (Odds Ratio [OR] per R allele 0.98[0.84–1.15], p = 0.8). Using conditional logistic regression, quartiles of PON1-activity showed a modest inverse relation with CAD risk (OR for the highest versus the lowest quartile 0.77[0.63–0.95], p = 0.01; p-trend = 0.06). PON1-activity adjusted for Q192R polymorphism correlated better with HDL-cholesterol (r = 0.26, p<0.0001) and more linearly predicted CAD risk (0.79[0.64–0.98], p = 0.03; p-trend = 0.008). However, these relationships were abolished after adjustment for HDL (particles-cholesterol-size) and apolipoproteinA-I (0.94[0.74–1.18], p-trend = 0.3).

**Conclusions/Significance:**

This study, shows that PON1-activity inversely relates to CAD risk, but not independent of HDL, due to its close association with the HDL-particle. These data strongly suggest that a low PON1-activity is not a causal factor in atherogenesis.

## Introduction

Oxidation of low-density lipoprotein (LDL) and the subsequent generation of lipid hydroperoxides has been shown to accelerate the development of atherosclerosis and to adversely affect cardiovascular outcome [1]–[3]. Consequently, a robust number of antioxidants that potently inhibit LDL-oxidation *in vitro* [4], [5] have been tested in clinical trials of impressive size, but most of these studies have yielded negative results [6]–[9]. Consequently, attention has shifted towards other physiological targets that may play a role in LDL-oxidation *in vivo*. In this respect, paraoxonase1 (PON1), a plasma enzyme that resides on the high-density lipoprotein (HDL) particle, has been shown to inhibit LDL-oxidation and is therefore also a strong candidate as a protective enzyme in the development of atherosclerosis [10]–[12].

Interestingly, two genetic variants of PON1, the Q192R and L55M polymorphisms (rs662 and rs854560, respectively) profoundly affect the activity of serum paraoxonase. The PON1-192 QQ gene product exhibits low hydrolytic activity towards paraoxon, whereas that of the RR-genotype shows high activity towards paraoxon [13], [14]. Conversely, the isoenzyme corresponding to this RR-genotype exhibits low hydrolytic activity towards lipid hydroperoxides. Likewise, gene products of the PON1-55 LL-genotype, LM-genotype and MM-genotype exhibit high, intermediate and low hydrolytic activity towards paraoxon, respectively. Although many association studies have reported on PON1-gene polymorphisms and CAD risk, these studies have yielded conflicting results; these reports were statistically underpowered, studied different ethnic and patient populations, utilized different sampling strategies as well as different genotyping procedures. These impediments have been discussed in a meta-analysis of association studies on PON1-genotype and CAD risk [15]. In contrast to PON1-genotype, only few studies have assessed the relationship between PON1-activity and CAD risk. In a cross-sectional study, hydrolysis rates of PON1 towards paraoxon and diazoxon were significantly lower in people with carotid disease than in controls [13]. Consistently, the prospective Caerphilly study showed that low PON1-activity towards paraoxon was associated with an increased risk of future CAD [14]. In line, PON1 activity and concentration were significantly lower in patients with severe coronary artery stenosis [16], whereas another study found no association between PON1 activity and coronary artery calcification [17]. In addition to this, elevated levels of PON1 activity healthy middle-aged women were shown to be associated with increased CAD risk [18]. These conflicting data have made it difficult to fully comprehend the role of PON1 in atherosclerosis. In fact, it remains uncertain whether the relation between PON1-activity and CAD risk is causal and whether it is independent of other CAD risk factors such as HDL cholesterol [15]. We used the concept of Mendelian randomization to evaluate whether PON1-activity may play a causal role in determining CAD risk [19]. We quantified the activity of PON1 towards paraoxon, determined two prominent genetic variants affecting PON1-activity, and assessed the consistency and proportionality of relationships between genotype, phenotype and risk of future CAD. We studied these relationships in a case-control study nested in the large, prospective EPIC (European Prospective Investigation into Cancer and Nutrition)–Norfolk study cohort.

## Results

Serum samples from 1138 cases and 2237 controls were included in the analysis from which 1099 cases were matched to two controls and 39 cases were matched to one control. The Q192R and L55M polymorphisms were available for 1077 cases (61 missing cases). The numbers in tables 1, 2, 3 may not add up to the total number because of missing data. Matching ensured that the distribution of age and sex was comparable between cases and controls. As expected, individuals who developed CAD during follow-up were more likely than controls to smoke and to have diabetes mellitus (Table 1). The use of alcohol was comparable between cases and controls. Likewise, in both men and women, levels of total cholesterol, LDL-cholesterol, triglycerides (non-fasting), apoB, systolic and diastolic blood pressure, BMI, waist circumference and CRP were significantly higher in cases than in controls, whereas HDL-cholesterol and apoA-I were significantly lower in cases than controls. Serum PON1-activity was similar in cases and controls (60.7±45.3 versus 62.6±45.8 U/L, P = 0.3). Sex-specific analyses revealed similar results. Interestingly, women had higher PON1-activity than men, consistent with higher HDL cholesterol and apoA-I levels in women (see data supplement for sex-specific data: Table S1). The distribution of PON1-Q192R and PON1-L55M polymorphisms did not differ between cases and controls (Table 1).

**Table 1:** Baseline characteristics of study participants.

	Controls	Cases	P
Total no. of patients, n	2237	1138	
Age, years	65±8	65±8	Matched
Women, n (%)	768 (40)	384 (40)	Matched
Body mass index, kg/m2	26.3±3.5	27.3±3.9	<0.0001
Waist circumference, cm	91±11	94±12	<0.0001
Smoking	–	–	<0.0001
-Never smoked, n	894	361	–
-Previous smoker, n	1138	592	–
-Current smoker, n	181	172	–
Alcohol use, units/week	7.4±9.5	6.6±10.0	0.07
Vitamin supplement use, n (%)	964 (43)	476 (42)	0.5
Vitamin C, µmol/l	52±20	46±20	<0.0001
Diabetes, n (%)	41 (2)	75 (7)	<0.0001
Systolic blood pressure, mmHg	139±18	144±19	<0.0001
Diastolic blood pressure, mmHg	84±11	86±12	<0.0001
Total cholesterol, mmol/l	6.3±1.2	6.5±1.2	<0.0001
LDL-cholesterol, mmol/l	4.1±1.0	4.3±1.0	<0.0001
HDL-cholesterol, mmol/l	1.37±0.40	1.27±0.37	<0.0001
Triglycerides, mmol/l	1.6 (1.1–2.7)	1.9 (1.2–3.3)	<0.0001
Apolipoprotein B, mg/dl	129±31	139±34	<0.0001
Apolipoprotein A-I, mg/dl	162±29	155±29	<0.0001
HDL size, nm	8.92±0.48	8.82±0.46	<0.0001
HDL particle number, nmol/L	34.2±5.5	33.3±5.9	<0.0001
C-reactive protein, mg/l	1.5 (0.9–3.4)	2.4 (1.0–5.3)	<0.0001
Myeloperoxidase, pmol/l	534 (323–855)	607 (348–858)	<0.0001
Paraoxonase-1 activity, U/L	62.6±45.8	60.7±45.3	0.3
PON1-192 genotype			0.9
-PON1-192 QQ, n	1092	548	–
-PON1-192 QR, n	847	415	–
-PON1-192 RR, n	177	92	–
PON1-55 genotype			0.4
-PON1-55 LL, n	869	424	–
-PON1-55 LM, n	932	486	–
-PON1-55 MM, n	263	140	–

Data are presented as mean (±SD) or number (percentage). Data for C-reactive protein, myeloperoxidase and triglycerides are presented as median (interquartile range). P-values are for mixed effects model with continuous variables, and for conditional logistic regression with dichotomous variables. LDL = low-density lipoprotein, HDL = high-density lipoprotein cholesterol.

**Table 2:** Associations between PON1 activity, PON1 genotype and risk factors.

	R (activity)	P *	192QQ	192QR	192RR	P^†^	55LL	55LM	55MM	P^†^
No. of subjects, n	3375		1640	1262	269		1293	1418	403	
PON1-activity, U/l	–	–	27±9	87±27	152±45	<0.0001	84±51	54±35	23±14	<0.0001
HDL-chol, mmol/l	0.156	<0.0001	1.33±0.40	1.34±0.39	1.34±0.38	0.8	1.34±0.39	133±0.39	1.29±0.37	0.1
HDL-particles, nmol/l	0.191	<0.0001	33.7±5.5	33.9±5.9	34.3±5.5	0.4	33.8±5.7	34.1±5.6	33.2±5.5	0.1
HDL-size, nm	0.059	<0.0001	8.9±0.5	8.9±0.5	8.9±0.5	0.9	8.9±0.5	8.9±0.5	8.9±0.5	0.3
LDL-chol, mmol/l	0.067	0.001	4.1±1.0	4.1±1.0	4.2±1.0	0.7	4.1±1.0	4.1±1.0	4.1±1.0	0.9
TG, mmol/l	0.013	0.6	1.7 (1.1–3.0)	1.7 (1.1–2.9)	1.8 (1.1–3.1)	0.4	1.7 (1.1–3.0)	1.7 (1.1–2.9)	1.7 (1.1–3.1)	0.9
ApoA-I, mg/dl	0.123	<0.0001	160±29	160±30	160±28	0.9	160±30	160±29	157±28	0.2
ApoB, mg/dl	0.026	0.4	132±32	132±33	133±32	0.9	132±33	132±32	134±33	0.7
CRP, mg/l	−0.041	0.05	1.7 (1.0–4.6)	1.7 (1.0–4.6)	2.1 (1.0–5.4)	0.7	1.7 (1.0–4.8)	1.7 (1.0–4.6)	1.7 (1.0–4.5)	0.8
MPO, pmol/l	−0.059	<0.0001	560 (343–805)	562 (345–886)	532 (312–883)	0.4	557 (325–885)	560 (354–890)	555 (310–897)	0.8
Alcohol use, units/wk	0.029	0.5	7.3±10.0	6.9±9.5	7.6±9.9	0.4	7.1±10.0	7.2±9.8	7.6±9.5	0.2
Vitamin-C, µmol/l	0.056	0.010	50±20	49±20	50±19	0.3	50±20	49±20	51±19	0.2

Data are presented as mean (±SD) or number (percentage). Data for C-reative protein (CRP), myeloperoxidase (MPO) and triglycerides (TG) are presented as median (interquartile range). R indicates two-tailed Pearsons (parametric) or Spearman's (non-parametric e.g. CRP, MPO and TG) correlation between PON1-activity and risk factors with the corresponding p-value (P*). Associations between PON1-genotype and risk factors are indicated by the p-values between groups (P^†^) from a one-way ANOVA.

**Table 3:** Distribution of cardiovascular risk factors by quartiles of PON1 activity.

PON1- activity (U/l)	1^st^ quartile	2^nd^ quartile	3^rd^ quartile	4^th^ quartile	P	R	P^†^
Variable	(<25.9)	(25.9–43.0)	(43.0–89.9)	(>89.9)			
Total no. of patients	844	844	844	843	–	–	–
Age, years	65.9±7.6	65.3±7.8	65.3±7.8	64.8±7.8	0.03	−0.058	<0.0001
Women, n (%)	248 (29)	342 (41)	281 (33)	368 (44)	<0.0001*	0.083	<0.0001
Body mass index, kg/m2	26.7±3.7	26.6±3.6	26.6±3.6	26.7±3.7	0.9	0.001	0.9
Waist circumference, cm	93.5±11.6	91.5±11.7	92.6±11.8	91.6±11.4	0.001	−0.043	0.01
Cigarette smoking					0.09*	–	–
-Never smoked, n	296	322	305	332	–	–	–
-Previous smoker, n	440	442	425	423	–	–	–
-Current smoker, n	98	70	103	82	–	–	–
Alcohol use, units/week	7.0±9.7	7.3±9.7	6.7±9.8	7.4±9.7	0.3	0.029	0.1
Vitamin supplement use, n (%)	342 (41)	377 (45)	360 (43)	361 (43)	0.4*	–	–
Plasma Vitamin C, µmol/l	48.8±20.0	51.6±20.1	47.6±19.6	52.1±±19.2	<0.0001	0.056	<0.0001
Diabetes mellitus, n (%)	39 (5)	28 (4)	30 (3)	19 (3)	0.07*	–	–
Systolic blood pressure, mmHg	140±18	141±19	141±18	141±18	0.9	0.009	0.6
Diastolic blood pressure, mmHg	84±12	85±±12	85±11	84±11	0.8	0.012	0.5
Total cholesterol, mmol/l	6.2±1.1	6.4±1.1	6.2±1.2	6.6±1.2	<0.0001	0.112	<0.0001
LDL-cholesterol, mmol/l	4.1±1.0	4.2±±1.0	4.1±1.0	4.3±1.0	<0.0001	0.067	<0.0001
HDL-cholesterol, mmol/l	1.24±0.37	1.39±±0.39	1.30±0.38	1.43±0.40	<0.0001	0.156	<0.0001
Triglycerides, mmol/l	1.8 (1.1–2.4)	1.7 (1.1–2.3)	1.7 (1.1–2.5)	1.7 (1.1–2.4)	0.4	0.013	0.5
Apolipoprotein B, mg/dl	132±32	132±31	131±34	135±32	0.1	0.026	0.2
Apolipoprotein A-I, mg/dl	155±29	162±28	155±29	166±30	<0.0001	0.123	<0.0001
HDL size, nm	8.8±0.5	8.9±0.5	8.9±0.5	8.9±0.5	<0.0001	0.059	0.001
HDL particle number, nmol/L	32.4±5.3	34.5±5.4	33.0±5.7	35.6±5.7	<0.0001	0.191	<0.0001
C-reactive protein, mg/l	1.9 (0.9–4.2)	2.1 (1.0–4.7)	1.8 (1.0–4.6)	1.6 (1.0–4.2)	<0.0001	−0.041	0.02
Myeloperoxidase, pmol/l	607 (342–802)	531 (337–825)	573 (325–751)	514 (340–762)	<0.0001	−0.059	0.001
Paraoxonase activity, U/L	19.9±4.1	32.5±4.6	86.5±13.6	127.0±34.7	–	–	–

Values are mean (±SD) or number (percentage). Data for C-reactive protein, meyloperoxidase and triglycerides are presented as median (±interquartile range). P = p-value for linearity between quartiles of PON1-activity and risk factor levels; R = Pearson's (parametric variables) or Spearman correlation (non-parametric variables e.g. C-reactive protein, meyloperoxidase and triglycerides) between PON1-activity and risk factors, and the corresponding p-value (P^†^). LDL = low-density lipoprotein, HDL = high-density lipoprotein. * indicates p-value by chi square test for dichotomous variables.

Table 2 shows the correlations between PON1-activity and risk factors and associations between both PON1-genotypes and risk factors. In brief, PON1-activity was positively correlated with HDL-particle number, HDL-cholesterol, HDL-size, apoA-I, LDL-cholesterol and vitamin-C concentration. There was a negative but weak correlation with levels of CRP and MPO. Similar trends were found among men and women in the sex-specific analyses (see data supplement for sex-specific data: Table S2).

**Figure 1:**
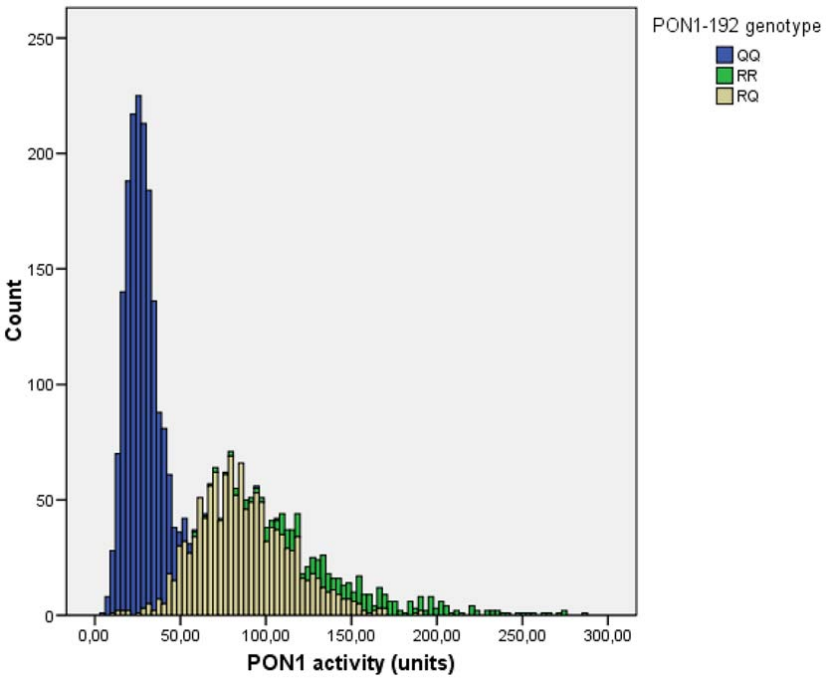
Histogram showing the triphasic distribution of PON1-activity according to the PON1-Q192R polymorphism.

**Figure 2:**
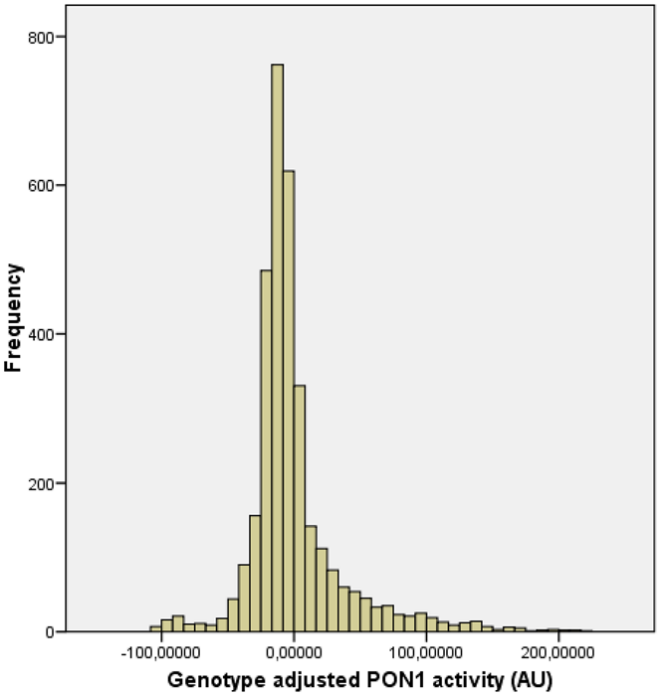
Histogram showing the normal distribution of PON1-activity after adjusting for the PON1-Q192R polymorphism.

Although both genotypes were significantly associated with PON1-activity, we found a stronger association for the PON1-Q192R polymorphism. Carriership of more PON1-192R alleles or more PON1-55M alleles was associated with a higher serum PON1-activity, concordant with a gene-dosing effect. Distribution of PON1-activity over genotypes was triphasic according to the PON1-Q192R polymorphism, illustrating the strong influence of this variant on the PON1-activity phenotype (Figure 1). Statistical adjustment for the PON1-Q192R polymorphism yielded a normally distributed PON1-activity (Figure 2), which serves as a proxy for PON1-concentration. PON1-activity adjusted for the PON1-Q192R polymorphism was similar in cases and controls (0.49±1.09 versus 0.76±0.79 AU/L, P = 0.8).

Table 3 shows the distribution of cardiovascular risk factors as stratified by PON1-activity quartiles (Table 3) and the PON1-Q192R polymorphism adjusted PON1-activity quartiles (Table 4). In brief, in both men and women, the PON1-Q192R polymorphism adjusted PON1-activity showed a strong association with HDL-cholesterol, HDL-particle number and apoA-I. There was a modest inverse, but significant linear relationship between the PON1-Q192R polymorphism adjusted PON1-activity and waist circumference, CRP, MPO and age, whereas a modest positive linear relationship was observed for gender, diabetes mellitus, total cholesterol, LDL-cholesterol, plasma vitamin-C concentration and HDL-size. Similar, although weaker, associations were found for the unadjusted PON1-activity quartiles (Table 4).

**Table 4:** Distribution of cardiovascular risk factors by quartiles of PON1-activity adjusted for PON1-192 genotype.

PON1-activity adjusted for PON1-192 genotype (AU/l)	1^st^ quartile	2^nd^ quartile	3^rd^ quartile	4^th^ quartile	P	R	P^†^
Variable	(<−16.8)	(−16.8 to –7.8)	(−7.8 to 5.6)	(>5.6)			
Total no. of patients	856	817	812	826	–	–	–
Age, years	66.2±7.4	65.2±8.0	65.0±7.8	64.8±7.9	0.001	−0.066	<0.0001
Women, n (%)	239 (34)	259 (48)	338 (38)	360 (51)	<0.0001*	0.144	<0.0001
Body mass index, kg/m2	26.7±3.6	26.7±3.7	26.5±3.6	26.7±3.7	0.7	−0.007	0.7
Waist circumference, cm	93.5±11.2	93.0±11.7	91.2±11.9	91.7±11.4	<0.0001	−0.073	<0.0001
Cigarette smoking					0.1*	–	–
-Never smoked, n	292	299	314	322	–	–	–
-Previous smoker, n	447	418	411	422	–	–	–
-Current smoker, n	109	88	76	77	–	–	–
Alcohol use, units/week	6.1±7.9	7.1±9.8	7.4±10.3	7.9±10.6	0.001	0.041	0.02
Vitamin supplement use, n (%)	344 (43)	333 (45)	380 (50)	360 (47)	0.03*	–	–
Plasma Vitamin C, µmol/l	49.9±19.9	51.7±20.2	47.7±19.5	52.0±19.1	<0.0001	0.104	<0.0001
Diabetes mellitus, n (%)	40 (5)	27 (4)	26 (3)	20 (3)	0.08*	–	–
Systolic blood pressure, mmHg	141±18	140±19	141±18	141±18	0.4	−0.003	0.8
Diastolic blood pressure, mmHg	84±11	84±12	85±11	84±11	0.4	−0.002	0.9
Total cholesterol, mmol/l	6.1±1.1	6.3±1.1	6.5±1.2	6.6±1.3	<0.0001	0.165	<0.0001
LDL-cholesterol, mmol/l	4.0±1.0	4.1±1.0	4.2±1.0	4.2±1.0	<0.0001	0.086	<0.0001
HDL-cholesterol, mmol/l	1.21±0.35	1.29±0.36	1.40±0.40	1.46±0.41	<0.0001	0.257	<0.0001
Triglycerides, mmol/l	1.7 (1.1–3.0)	1.7 (1.1–2.9)	1.7 (1.1–2.9)	1.8 (0.5–3.1)	0.5	0.008	0.6
Apolipoprotein B, mg/dl	132±34	132±31	132±32	134±33	0.5	0.027	0.1
Apolipoprotein A-I, mg/dl	152±29	157±28	162±28	168±30	<0.0001	0.208	<0.0001
HDL size, nm	8.8±0.5	8.9±0.5	8.9±0.5	8.9±0.5	<0.0001	0.120	<0.0001
HDL particle number, nmol/L	32.0±5.5	33.0±5.1	34.5±5.4	36.0±5.7	<0.0001	0.269	<0.0001
C-reactive protein, mg/l	2.0 (1.2–6.0)	1.7 (1.1–4.6)	1.5 (1.0–3.9)	1.7 (0.1–4.4)	<0.0001	−0.059	0.001
Myeloperoxidase, pmol/l	626 (14–1238)	576 (24–1118)	523 (25–1015)	515 (25–1004)	<0.0001	−0.103	<0.0001
Paraoxonase activity, U/L	40.6±23.7	35.8±22.8	50.1±25.3	121.8±42.2	<0.0001	0.665	<0.0001

Values are mean (±SD) or number (percentage). Data for C-reative protein, meyloperoxidase and triglycerides are presented as median (±interquartile range). P = p-value for linearity between PON1-192 genotype adjusted PON1-activity quartiles and risk factor levels; R = Pearson's (parametric variables) or Spearman correlation (non-parametric variables e.g. C-reactive protein, meyloperoxidase and triglycerides) between PON1-192 genotype adjusted serum PON1 activity levels and risk factors, and the corresponding p-value (P^†^). LDL = low-density lipoprotein, HDL = high-density lipoprotein. * indicates p-value by chi square test for dichotomous variables.

Table 5A shows the unadjusted and adjusted ORs for future CAD by quartiles of PON1-activity. Serum PON1-activity was modestly, although not linearly related to risk of future CAD. Adjustment for HDL-parameters (HDL-cholesterol, HDL-particle number, HDL-size and apoA-I), did not considerably affect the risk estimate. Further adjustment for risk factors (alcohol use, BMI, CRP, diabetes mellitus, fasting time, LDL-cholesterol, MPO, smoking, systolic blood pressure, plasma vitamin-C, vitamin supplement use, waist circumference and triglycerides (non-fasting) did not substantially alter the observed relationship.

**Table 5:** Risk of future coronary artery disease by quartiles of PON1 activity and by quartiles of PON-1 activity adjusted for PON1-192 genotype.

A					
PON1 activity, U/l	1^st^ quartile	2^nd^ quartile	3^rd^ quartile	4^th^ quartile	P
	(<25.9)	(25.9–43.0)	(43.0–89.9)	(>89.9)	
Cases / controls	322 / 844	258 / 844	284 / 844	274 / 843	
OR unadjusted	1.0	0.70 (0.57–0.86)	0.81 (0.67–0.99)	0.77 (0.63–0.95)	0.06
OR adjusted for HDL parameters, (1)	1.0	0.74 (0.57–0.94)	0.83 (0.65–1.05)	0.79 (0.61–1.02)	0.1
OR adjusted for (1) + risk factors, (2)	1.0	0.73 (0.55–0.98)	0.83 (0.63–1.09)	0.78 (0.58–1.05)	0.2

B					
PON1- activity adjusted for PON1-192 genotype, AU/l	1^st^ quartile	2^nd^ quartile	3^rd^ quartile	4^th^ quartile	P
	(<−16.8)	(−16.8 to−7.8)	(−7.8 to 5.6)	(>5.6)	
Cases / controls	310 / 856	291 / 817	249 / 812	260 / 826	
OR unadjusted	1.0	0.95 (0.77–1.17)	0.76 (0.61–0.94)	0.79 (0.64–0.98)	0.008
OR adjusted for HDL parameters, (1)	1.0	0.99 (0.80–1.24)	0.85 (0.67–1.06)	0.94 (0.74–1.18)	0.3
OR adjusted for (1) + risk factors, (2)	1.0	1.00 (0.80–1.44)	0.76 (0.61–1.13)	0.83 (0.68–1.28)	0.1

Odds ratios (ORs) for the risk of future CAD events by quartiles of PON1-activity and ORs by quartiles of PON1 activity adjusted for PON1-192 genotype. (1) Adjustment for HDL parameters (HDL-particle number, HDL-cholesterol, HDL-size and apoA-I), (2) Adjustment for HDL parameters and risk factors (alcohol use, BMI, CRP, diabetes mellitus, fasting time, LDL cholesterol, myeloperoxidase, smoking, systolic blood pressure, vitamin C, vitamin supplement use, waist circumference, triglycerides). P = p-value for trend = χ^2^ linear trend with 1 degree of freedom. AU indicates arbitrary units.

Table 5B shows, however, that the PON1-Q192R polymorphism adjusted PON1-activity was strongly inversely related with the risk of future CAD (OR 0.79 for the highest versus the lowest quartile, 95% CI 0.64 to 0.98, *P* for linearity = 0.008). This relation was abolished by adjustment for HDL related parameters (HDL-cholesterol, HDL-particle number, HDL-size and apoA-I). Additional adjustments for cardiovascular risk factors did not affect the results. Similar trends were found among men and women in the gender-specific analyses (see data supplement for gender-specific data: Table S3).

Backward stepwise regression analysis for PON1-activity, PON1-genotype as well as PON1-activity adjusted for PON1-192 and PON1-55 genotype revealed no significant effect of these variables and CAD risk (see data supplement: Table S4, S5, S6).

Haplotypes of PON1-192 and PON1-55 genotypes where equally distributed between cases and controls. Further the effect of these different haplotypes on PON1 activity, PON1-genotypes and PON1-activity adjusted for PON1-192 genotype are reported in the supplementary table (see data supplement: Table S7). No significant relation between combined haplotypes and CAD risk was found.

## Discussion

In this large prospective case-control study among apparently healthy men and women, we observed that PON1-activity was modestly and inversely related with the risk of future CAD. However, given the fact that the Q192R polymorphism strongly affects lifelong PON1-activity, the absence of a relation between the Q192R polymorphism and CAD risk suggests that PON1-activity is not a causal factor in atherogenesis. The apparent protective function of PON1 can only be appreciated against the background of its close association with HDL.

Whereas PON1-L55M genotype was modestly associated with PON1-activity in our study, the PON1-Q192R polymorphism was the strongest determinant of PON1-activity against paraoxon. Although higher PON1-activity against paraoxon tended to be associated with a lower CAD risk, this relation was not linear. More importantly, PON1-Q192R genotype had no effect on CAD risk. In the context of Mendelian randomization [19], these findings suggest that the relation between PON1-activity against paraoxon and CAD risk is not causal. Had this been the case, one would expect consistent and proportional relationships among genotype, phenotype and CAD risk. We observed that carriership of a 192R allele was associated with a 62.5 U/l increase in PON1-activity per allele. In turn, an increase of 62.5 U/l in PON1-activity corresponds approximately to the difference between the first and third PON1 activity quartile, which was associated with an OR for CAD of 0.81(95% CI [0.67–0.99]). However, carriership of a 192R allele was not associated with a proportional decrease in CAD risk. In fact, we observed a null relationship (OR 0.98[0.84–1.15], p = 0.8). The present findings imply that previous findings of an inverse relation between PON1 activity and CAD risk were troubled by confounding. In this respect, several issues deserve closer attention. PON1 is located predominantly on HDL-particles. Since HDL has a strong inverse relation to CAD risk [24], other protective effects mediated by or associated with HDL [25] are likely to contribute to the PON1–CAD relation. People with more circulating HDL-particles can be expected to have a higher plasma PON1-concentration. Because PON1-activity is a function of PON1-concentration and PON1-genotype [26]–[27], we could obtain a proxy for PON1-concentration by statistically adjusting PON1-activity for the PON1-Q192R polymorphism. Indeed, the PON1-Q192R polymorphism adjusted PON1-activity correlated better with HDL-related parameters than unadjusted PON1-activity did. Similarly, it was a better predictor of CAD risk than unadjusted PON1-activity. However, statistical adjustment for HDL-related parameters abolished the inverse relation between PON1-activity adjusted for the PON1-Q192R polymorphism and CAD risk in the present cohort (Table 4B). These findings lend further support to the concept that the relation between PON1-activity and CAD risk to a relevant extent confounded by HDL-cholesterol levels and is not based on causality.

### PON1 and CAD risk

Numerous studies have reported associations between the PON1-Q192R and L55M polymorphisms and risk of CAD [28]–[34], whereas other studies found no such relation [35]–[39]. However, a meta-analysis reported no significant association for the L55M variant [15]. In addition, it observed that the significant association for the Q192R variant was largely explained by small studies while larger studies which are less prone to statistical bias, showed no significant association. Interestingly, recent genome-wide association studies support our findings and have not revealed any associations between the PON1-genotype and risk of CAD [40], [41]. In addition, Jarvik et al. have shown that PON1-activity towards paraoxon is a better predictor of cardiovascular risk than PON1 genotype [13]. In the present study, we substantiate this finding, since no relationship was observed between the PON1-Q192R or PON1-L55M polymorphisms and risk of future CAD events. In this respect, it should be noted that the present study was a large prospective case-control cohort, comprising 1138 cardiovascular events, whereas other observational studies have used a small sample size and/or a retrospective design.

Finally, the role of inflammation in atherosclerosis has been substantiated in numerous studies. However, in the present study, inflammatory markers such as CRP and MPO did not affect the relation between PON1 and CAD risk. An explanation for this finding might be the modest relation between both CRP and MPO versus PON1 activity.

### PON1 and Vitamins

Vitamin C intake has previously been shown to be associated with increased PON1 activity [42]. In line, the present study demonstrated that PON1 activity is primarily genotype dependent, and is just modestly influenced by plasma vitamin C concentration. It has been suggested that a reduction in oxidative stress related to vitamin C intake may preserve PON1 activity. In addition, it has been shown that those subjects using vitamin supplements are more likely to engage in healthy lifestyles, of which the factors are multivariate [43]. Thus, it is possible that some factor correlated with vitamin intake is actually influencing PON1 activity.

### Study Limitations

The results of the present study should be considered in light of its potential limitations. First, case ascertainment is an issue in the design of every prospective study, including this one. However, a validation study indicated that case ascertainment in our study was at least equivalent to that of other large prospective cohort studies [44]. Second, the findings of the present study apply to Caucasians and should be carefully extrapolated to non-Caucasian populations, especially since PON1-genotype frequencies have been reported to vary substantially between Caucasian and East Asian populations (PON1-192RR homozygosity occurs in only about 10% of Caucasians, but in about 40% of East Asians). Also, activities of PON1 towards other substrates than paraoxon, e.g. lipid hydroperoxides, might have a distinctly different impact on atheroprotection. Therefore we cannot exclude that other tests for PON1 activity would yield different results. However, since the PON1-192 RR genotype, apart from high reactivity towards paraoxon, is also characterized by low reactivity towards lipid hydroperoxides, the absence of a relationship between the PON1-Q192R polymorphism and CAD risk suggests that this particular phenotype can not strongly modulate CAD risk either. Third, the in vitro hydrolysis of paraoxon reflecting PON1 activity may be a suboptimal method to measure its actual in vivo antioxidant capacity. Methods to more accurately PON1 in vivo antioxidant capacity are currently not at hand.

Fourth, a common limitation of case-control studies, even when they are of a prospective nature is that subjects who contract CAD at a future date are bound to have more classical risk factors than subjects who do not. This may limit the contribution of PON1-activity and genotype on CAD risk. Despite these limitations we tried to minimize the influence of these stronger risk factors on CAD risk to an acceptable degree (see stepwise backward regression analysis in supplementary tables S4-S6). Finally, the Mendellian randomization analyses in the present study should be interpreted with caution, since the PON1-activity and CAD risk association is nonlinear. The concept of Mendelian randomization is based on several assumptions, including a linear relation between the trait of interest and disease risk.

### Conclusion

We demonstrate a modest and inverse relationship between PON1-activity and risk of future CAD among apparently healthy men and women. The PON1-Q192R polymorphism strongly affects PON1-activity but was not associated with risk of future CAD. The inconsistency of the relationships among PON1-genotype, PON1-activity and CAD risk suggests that PON1 is not a causal factor in the development of CAD. Concurrently, we observed that the relationship between a proxy for PON1-concentration and CAD risk was abolished upon adjustment for HDL-related parameters, indicating that this relationship is confounded by HDL-cholesterol.

## Materials and Methods

The EPIC-Norfolk cohort study is a prospective population study of 25663 male and female inhabitants of Norfolk, United Kingdom, aged between 45 and 79 years old. EPIC-Norfolk is part of the 10-country collaborative EPIC study designed to investigate dietary and other determinants of cancer. Additional data were obtained to enable assessment of determinants of other diseases. Participants were enrolled between 1993 and 1997, completed a baseline questionnaire survey and attended a clinic visit. The study cohort was similar to UK population samples with regard to many characteristics, including anthropometry, blood pressure, and lipids, but with a lower proportion of smokers. Participants were recruited by mail from age-gender registers of general practices. At the baseline survey between 1993 and 1997, participants completed a detailed health and lifestyle questionnaire, and additional data collection was performed by trained nurses at a clinic visit as described previously. All individuals have been flagged for mortality at the UK Office of National Statistics, with vital status ascertained for the entire cohort. Death certificates of individuals enrolled in the study were used to classify the deaths. The death certificates were coded by trained nosologists according to the International Classification of Diseases (ICD) 9^th^ revision. Death was considered due to CAD if the underlying cause was coded as ICD 410 to 414. In addition, participants admitted to a hospital were identified by their unique National Health Service number by data linkage with ENCORE (East Norfolk Health Authority database), which identifies all hospital contacts throughout England and Wales for Norfolk residents. Participants were identified as having CAD during follow-up if they had a hospital admission and/or died with CAD listed as an underlying cause. We report results with follow-up up to January 2003, an average of 6 years. The Norwich District Health Authority Ethics Committee approved the study, and all participants gave signed informed consent.

### Participants

For the present nested case-control study, we identified study participants who did not report a history of heart attack or stroke at the baseline clinic visit. A total of 1138 apparently healthy individuals developed fatal or nonfatal CAD during follow-up. A total of 2237 controls were apparently healthy study participants who remained free of CAD during follow-up. 1099 cases were matched to two controls and 39 cases were matched to one control by sex, age (within 5 years), and date of visit (within 3 months). Data on PON1-activity was available in all study subjects, but data on PON1-genotype was missing in 61 cases.

### Biochemical Analyses

Non-fasting blood samples were taken by vein puncture into serum tubes. Serum levels of total cholesterol, HDL cholesterol, and triglycerides were measured on fresh samples with the RA 1000 auto-analyzer (Bayer Diagnostics, Basingstoke, United Kingdom). LDL-cholesterol levels were calculated with the Friedewald formula [20]. Blood samples were also stored at minus 80° Celsius. Samples were later thawed and processed for analysis. Serum levels of apolipoproteinA-I (apoA-I) and B (apoB) were measured by rate immunonephelometry (Behring Nephelometer BNII, Marburg, Germany) with calibration traceable to the International Federation of Clinical Chemistry primary standards [21]. The interassay coefficients of variation of the apoA-I and apoB measurements were 5% and 3%, respectively. HDL-particle number and HDL-size were measured with an automated nuclear magnetic resonance spectroscopic assay as described previously [22]. C-reactive protein (CRP) levels were measured as described previously [23]. Serum PON1-activity toward paraoxon was analyzed as previously described [14]. Serum concentration of myeloperoxidase (MPO) was measured by use of a commercially available ELISA (CardioMPO Test, Prognostix, Cleveland, Ohio). Samples were analyzed in random order to avoid systematic bias. Researchers and laboratory personnel had no access to identifiable information and could identify samples by number only.

### PON1 Genotyping

We investigated the two most common single nucleotide polymorphisms (SNP) in the PON1-gene; two substitutions in PON1 [Q192R, rs662, within exon 6 of the PON1 locus, and L55M, rs854560, within exon 3 of the PON1 locus) were analyzed. Genotyping for rs662 and rs854560 was conducted by KBioscience (http://www.kbioscience.co.uk) using KASPar technology. Cases and controls were randomly allocated across DNA study plates with two duplicate samples and two water controls in each 96-well plate. Concordancy between duplicate samples was 100% for both SNPs. Both SNPs were in Hardy-Weinberg equilibrium in control participants (P>0.4).

### Statistical Analysis

Baseline characteristics were compared between cases and controls with a mixed-effects model for continuous variables or conditional logistic regression for categorical variables. Triglycerides (non-fasting) and CRP levels had a skewed distribution, and therefore both variables were log-transformed before statistical analysis. Since the PON1-Q192R polymorphism affects serum PON1-activity, but not its concentration, we adjusted PON1-activity for the PON1-Q192R genotype to obtain a proxy for PON1-concentration. Analyses were performed for both (unadjusted) PON1-activity and PON1-activity adjusted for the PON1-Q192R polymorphism.

Associations between PON1-genotype, PON1-activity and cardiovascular risk factors were assessed with Pearson correlations for parametric variables and Spearman correlations for non-parametric variables.

Subsequently, PON1-activity and PON1-activity adjusted for the PON1-Q192R polymorphism were categorized into (sex-specific) quartiles based on the distribution in the controls. Mean levels of cardiovascular risk factors were calculated per quartile. Further, conditional logistic regression was used to calculate odds ratios (ORs) and corresponding 95% confidence intervals (CIs) per quartiles of PON1-activity (±adjustment for PON1-Q192R), as an estimate of the relative risk of CAD with the lowest quartile as the reference category. Conditional logistic regression took into account the matching for sex, age and enrollment time, and ORs were additionally adjusted for alcohol use, apolipoprotein A-I, body mass index (BMI), CRP, diabetes mellitus, fasting time, HDL-cholesterol, HDL-particle number, HDL-size, LDL-cholesterol, MPO, smoking, systolic blood pressure, plasma vitamin-C, vitamin supplement use, waist circumference and non-fasting triglycerides. ORs were also calculated after additional adjustment for PON1-L55M genotype. Finally, we performed stepwise (backward) cox regression analysis for PON1-activity, PON1-genotype as well as PON1-activity adjusted for PON1-genotype to predict the best model for risk of CAD. Statistical analyses were performed with SPSS software (version 12.0.1).

## Supporting Information

Table S1 Sex-specific characteristics of study participants.
(0.08 MB DOC)

Table S2 Sex-specific associations of PON1 activity, PON1 genotype and risk factors.
(0.07 MB DOC)

Table S3 Sex-specific odds ratios of future coronary artery disease by quartiles of PON1 activity and by quartiles of PON-1 activity adjusted for PON1-192 genotype.
(0.06 MB DOC)

Table S4 Backward Stepwise Cox Regression Analysis: HDL-cholesterol with all and excluded variables.
(0.08 MB DOC)

Table S5 Backward Stepwise Cox Regression Analysis: HDL-particles with all and excluded variables.
(0.08 MB DOC)

Table S6 Backward Stepwise Cox Regression Analysis: apolipoproteinA-I with all and excluded variables.
(0.08 MB DOC)

Table S7 Distribution of combined PON1-haplotypes and its effect on HDL-cholesterol, HDL-particles, PON1-acitivity and PON1-activity adjusted for PON1-192 genotype.
(0.04 MB DOC)
