# Supplementary material for: Both Paraoxonase-1 Genotype and Activity Do Not Predict the Risk of Future Coronary Artery Disease; the EPIC-Norfolk Prospective Population Study
Source: PLoS One. 2009 Aug 27;4(8):e6809. doi: 10.1371/journal.pone.0006809 (PMC2728540; doi:10.1371/journal.pone.0006809)
Supplement: Table S1 — Sex-specific characteristics of study participants. (0.08 MB DOC) [file pone.0006809.s001.doc]

**Table S1. Sex-specific characteristics of study participants**

| **A** | **Controls** | **Cases** | **P** |
| --- | --- | --- | --- |
| **Men, n** | 1411 | 725 |  |
| Age, years | 64 ± 8 | 65 ± 8 | Matched |
| Body mass index, kg/m2 | 26.4 ± 3.1 | 27.2 ± 3.5 | <0.0001 |
| Waist circumference, cm | 96 ± 9 | 98 ± 10 | <0.0001 |
| Smoking | - | - | <0.0001 |
| -Never smoked, n | 441 | 176 | - |
| -Previous smoker, n | 835 | 432 | - |
| -Current smoker, n | 118 | 110 | - |
| Alcohol use, units/week | 9.4 ± 10.9 | 8.5 ± 11.6 | 0.1 |
| Vitamin supplement use, n (%) | 545 (39) | 284 (39) | 0.7 |
| Vitamin C, µmol/l | 48 ± 18 | 43 ± 19 | <0.0001 |
| Diabetes, n (%) | 33 (2) | 53 (7) | <0.0001 |
| Systolic blood pressure, mmHg | 139 ± 17 | 145 ± 18 | <0.0001 |
| Diastolic blood pressure, mmHg | 85 ± 11 | 87 ± 12 | <0.0001 |
| Total cholesterol, mmol/l | 6.1 ± 1.1 | 6.3 ± 1.1 | <0.0001 |
| LDL-cholesterol, mmol/l | 4.0 ± 0.9 | 4.1 ± 1.0 | <0.0001 |
| HDL-cholesterol, mmol/l | 1.25 ± 0.33 | 1.16 ± 0.31 | <0.0001 |
| Triglycerides, mmol/l | 1.7 (0.5 - 2.9) | 2.0 ± (0.5 – 3.5) | <0.0001 |
| Apolipoprotein B, mg/dl | 128 ± 30 | 137 ± 33 | <0.0001 |
| Apolipoprotein A-I, mg/dl | 153 ± 25 | 148 ± 26 | <0.0001 |
| HDL size, nm | 8.79 ± 0.42 | 8.72 ± 0.43 | <0.0001 |
| HDL particle number, nmol/L | 33.4 ± 5.3 | 32.3 ± 5.7 | <0.0001 |
| C-reactive protein, mg/l | 1.4 (0.4 - 3.6) | 2.2 (0.6 - 5.7) | <0.0001 |
| Myeloperoxidase, pmol/l | 559 (354 – 906) | 640 (408 – 986) | <0.0001 |
| Paraoxonase-1 Activity, U/L | 59.9 ± 43.9 | 57.6 ± 43.7 | 0.3 |
| PON1-192 genotype |  |  | 0.1 |
| -PON1-192 QQ, n | 691 | 365 | - |
| -PON1-192 QR, n | 540 | 253 | - |
| -PON1-192 RR, n | 116 | 40 | - |
| PON1-55 genotype |  |  | 0.9 |
| -PON1-55 LL, n | 549 | 285 | - |
| -PON1-55 LM, n | 597 | 300 | - |
| -PON1-55 MM, n | 183 | 38 | - |
| **B** | **Cases** | **Controls** | **P** |
| **Woman, n** | 826 | 413 |  |
| Age, years | 67 ± 7 | 67 ± 7 | Matched |
| Body mass index, kg/m2 | 26.2 ± 4.1 | 27.3 ± 4.5 | <0.0001 |
| Waist circumference, cm | 83 ± 10 | 87 ± 11 | <0.0001 |
| Smoking | - | - | <0.0001 |
| -Never smoked, n | 453 | 185 | - |
| -Previous smoker, n | 303 | 160 | - |
| -Current smoker, n | 63 | 62 | - |
| Alcohol use, units/week | 3.9 ± 4.9 | 3.1 ± 4.9 | 0.01 |
| Vitamin supplement use, n (%) | 419 (51) | 192 (46) | 0.5 |
| Vitamin C, µmol/l | 59 ± 20 | 51 ± 20 | <0.0001 |
| Diabetes, n (%) | 8 (1) | 22 (5) | <0.0001 |
| Systolic blood pressure, mmHg | 138 ± 18 | 143 ± 19 | <0.0001 |
| Diastolic blood pressure, mmHg | 82 ± 11 | 85 ± 12 | <0.0001 |
| Total cholesterol, mmol/l | 6.6 ± 1.2 | 6.9 ± 1.3 | <0.0001 |
| LDL-cholesterol, mmol/l | 4.3 ± 1.1 | 4.5 ± 1.1 | <0.0001 |
| HDL-cholesterol, mmol/l | 1.58 ± 0.42 | 1.45 ± 0.39 | <0.0001 |
| Triglycerides, mmol/l | 1.5 (1.1 - 2.2) | 1.8 ± (1.3 – 2.6) | <0.0001 |
| Apolipoprotein B, mg/dl | 132 ± 33 | 142 ± 36 | <0.0001 |
| Apolipoprotein A-I, mg/dl | 177 ± 30 | 169 ± 30 | <0.0001 |
| HDL size, nm | 9.16 ± 0.47 | 8.99 ± 0.47 | <0.0001 |
| HDL particle number, nmol/L | 35.6 ± 5.6 | 35.0 ± 5.8 | <0.0001 |
| C-reactive protein, mg/l | 1.6 (0.8 - 3.5) | 2.6 (1.1 – 5.8) | <0.0001 |
| Myeloperoxidase, pmol/l | 505 (334 – 809) | 562 (345 – 871) | <0.0001 |
| Paraoxonase-1 Activity, U/L | 67.3 ± 48.6 | 66.2 ± 47.6 | 0.7 |
| PON1-192 genotype |  |  | 0.7 |
| -PON1-192 QQ, n | 401 | 183 | - |
| -PON1-192 QR, n | 307 | 162 | - |
| -PON1-192 RR, n | 61 | 33 | - |
| PON1-55 genotype |  |  | 0.1 |
| -PON1-55 LL, n | 320 | 139 | - |
| -PON1-55 LM, n | 335 | 186 | - |
| -PON1-55 MM, n | 80 | 46 | - |

Data are presented as mean (±SD) or number (percentage). Data for C-reative protein, myeloperoxidase and triglycerides are presented as median (interquartile range). P-values are for mixed effects model with continuous variables, and for conditional logistic regression with dichotomous variables. LDL = low-density lipoprotein, HDL = high-density lipoprotein cholesterol. A total of 1138 cases and 2237 controls were included in the analysis from which 1099 cases were matched to two controls and 39 cases were matched to one control. Data on PON1 genotype was missing in 61 cases. Values can be based on a lower number of subjects for some variables.
