# Supplementary material for: Both Paraoxonase-1 Genotype and Activity Do Not Predict the Risk of Future Coronary Artery Disease; the EPIC-Norfolk Prospective Population Study
Source: PLoS One. 2009 Aug 27;4(8):e6809. doi: 10.1371/journal.pone.0006809 (PMC2728540; doi:10.1371/journal.pone.0006809)
Supplement: Table S2 — Sex-specific associations of PON1 activity, PON1 genotype and risk factors. (0.07 MB DOC) [file pone.0006809.s002.doc]

**Table S2.** Sex-specific associations of PON1 activity, PON1 genotype and risk factors

|  | **R for activity** | **P*** | **192QQ** | **192QR** | **192RR** | **P^†^** | **55LL** | **55LM** | **55MM** | **P^†^** |
| --- | --- | --- | --- | --- | --- | --- | --- | --- | --- | --- |
| **Men, n** | 2136 | - | 1052 | 791 | 173 |  | 831 | 896 | 273 |  |
| PON1 activity, U/l | - | - | 26 ± 8 | 83 ± 27 | 146 ± 44 | <0.0001 | 84 ± 51 | 54 ± 35 | 23 ±14 | <0.0001 |
| HDL chol, mmol/l | 0.147 | <0.0001 | 1.22 ± 0.34 | 1.22 ± 0.33 | 1.24 ± 0.32 | 0.8 | 1.23 ± 0.33 | 122 ± 0.33 | 1.21 ± 0.33 | 0.6 |
| HDL particles, nmol/l | 0.171 | <0.0001 | 32.9 ± 5.4 | 33.1 ± 5.7 | 33.2 ± 5.0 | 0.9 | 33.0 ± 5.5 | 33.1 ± 5.3 | 32.5 ± 5.5 | 0.3 |
| HDL size, nm | 0.051 | 0.018 | 8.8 ± 0.4 | 8.8 ± 0.5 | 8.8 ± 0.4 | 0.8 | 8.8 ± 0.4 | 8.8 ± 0.4 | 8.8 ± 0.4 | 0.5 |
| LDL chol, mmol/l | 0.044 | 0.049 | 4.0 ± 0.9 | 4.0 ± 1.0 | 4.1 ± 1.0 | 0.5 | 4.0 ± 1.0 | 4.0 ± 1.0 | 4.0 ± 1.0 | 0.8 |
| Triglycerides, mmol/l | -0.004 | 0.9 | 1.8 (1.3–2.6) | 1.7 (1.3–2.5) | 1.8 (1.3-2.6) | 0.7 | 1.8 (1.3-2.5) | 1.8 (1.3-2.5) | 1.8 (1.3-2.7) | 0.6 |
| ApoA-I, mg/dl | 0.101 | <0.0001 | 152 ± 26 | 151 ± 26 | 152 ± 24 | 0.9 | 151 ± 26 | 152 ± 25 | 151 ± 28 | 0.8 |
| ApoB, mg/dl | 0.023 | 0.3 | 131 ± 30 | 131 ± 33 | 129 ± 31 | 0.8 | 130 ± 32 | 131 ± 31 | 135 ± 32 | 0.2 |
| CRP, mg/l | -0.041 | 0.058 | 1.6 (0.8-3.4) | 1.6 (0.7-3.5) | 2.1 (1.1-4.3) | 0.2 | 1.7 (0.8-3.9) | 1.5 (0.7-3.3) | 1.7 (0.8-3.3) | 0.3 |
| MPO, pmol/l | -0.073 | 0.001 | 588 (372-946) | 588 (374-938) | 543 (368-932) | 0.3 | 583 (363-950) | 573 (378-910) | 588 (365-1024) | 0.4 |
| Alcohol use, units/wk | -0.078 | <0.0001 | 9.3 ± 11.4 | 8.9 ± 10.9 | 9.7 ± 11.3 | 0.7 | 9.1 ± 11.5 | 9.4 ± 11.2 | 9.1 ± 10.4 | 0.4 |
| Vitamin C, µmol/l | 0.052 | 0.018 | 47 ± 19 | 46 ± 18 | 47 ± 18 | 0.7 | 46 ± 19 | 46 ± 18 | 49 ± 20 | 0.2 |
| **Woman, n** | 1239 | - | 580 | 466 | 94 |  | 457 | 519 | 124 |  |
| PON1 activity, U/l | - | - | 29 ± 10 | 94 ± 27 | 165 ± 43 | <0.0001 | 84 ± 51 | 54 ± 35 | 23 ±14 | <0.0001 |
| HDL chol, mmol/l | 0.122 | <0.0001 | 1.53 ± 0.42 | 1.55 ± 0.41 | 1.54 ± 0.41 | 0.9 | 1.55 ± 0.40 | 1.54 ± 0.42 | 1.47 ± 0.37 | 0.2 |
| HDL particles, nmol/l | 0.188 | <0.0001 | 35.2 ± 5.4 | 35.4 ± 6.0 | 36.4 ± 5.7 | 0.059 | 35.4 ± 5.7 | 35.5 ± 5.8 | 34.9 ± 5.0 | 0.5 |
| HDL size, nm | 0.007 | <0.0001 | 9.1 ± 0.5 | 9.1 ± 0.5 | 9.0 ± 0.5 | 0.2 | 9.1 ± 0.5 | 9.1 ± 0.5 | 9.1 ± 0.5 | 0.9 |
| LDL chol, mmol/l | 0.071 | 0.001 | 4.3 ± 1.1 | 4.4 ± 1.1 | 4.5 ± 1.1 | 0.3 | 4.4 ± 1.1 | 4.3 ± 1.1 | 4.3 ± 1.1 | 0.7 |
| Triglycerides, mmol/l | 0.007 | 0.8 | 1.6 (1.1–2.3) | 1.6 (1.1–2.3) | 1.8 (1.3-2.6) | 0.4 | 1.6 (1.1-2.4) | 1.6 (1.1-2.3) | 1.6 (1.2-2.4) | 0.9 |
| ApoA-I, mg/dl | 0.096 | <0.0001 | 174 ± 30 | 175 ± 32 | 174 ± 28 | 0.9 | 176 ± 30 | 175 ± 31 | 168 ± 25 | 0.2 |
| ApoB, mg/dl | 0.02 | 0.4 | 135 ± 34 | 135 ± 35 | 140 ± 35 | 0.5 | 136 ± 36 | 135 ± 33 | 134 ± 36 | 0.7 |
| CRP, mg/l | -0.037 | 0.2 | 1.8 (0.8-4.4) | 2.0 (0.9-4.2) | 2.1 (0.9-4.4) | 0.7 | 1.8 (0.9-3.9) | 2.1 (1.0-5.2) | 1.8 (0.9-4.6) | 0.7 |
| MPO, pmol/l | -0.024 | 0.4 | 520 (340-821) | 530 (335-793) | 501 (342-914) | 0.4 | 519 (347-805) | 538 (336-864) | 486 (326-735) | 0.7 |
| Alcohol use, units/wk | -0.015 | 0.5 | 3.8 ± 5.3 | 3.4 ± 4.6 | 3.5 ± 4.3 | 0.5 | 3.6 ± 4.7 | 3.4 ± 4.7 | 4.4 ± 5.8 | 0.2 |
| Vitamin C, µmol/l | 0.018 | 0.010 | 57 ± 20 | 55 ± 21 | 57 ± 20 | 0.2 | 57 ± 21 | 55 ± 21 | 55 ± 18 | 0.3 |

Data are presented as mean (±SD) or number (percentage). Data for C-reative protein (CRP), myeloperoxidase (MPO) and triglycerides are presented as median (interquartile range). Values may be based on a lower number of subjects for some variables because of missing data. R indicates two-tailed Pearsons (parametric) or Spearman’s (non-parametric e.g. CRP, MPO and triglycerides) correlation between PON1 activity and risk factors with the corresponding p-value (P*). Associations between PON1 genotype and risk factors are indicated by the p-values between groups (P^†^) from a one-way ANOVA. A total of 1138 cases and 2237 controls were included in the analysis from which 1099 cases were matched to two controls and 39 cases were matched to one control. Data on PON1 genotype was missing in 61 cases. Values can be based on a lower number of subjects for some variables.
