# Supplementary material for: Both Paraoxonase-1 Genotype and Activity Do Not Predict the Risk of Future Coronary Artery Disease; the EPIC-Norfolk Prospective Population Study
Source: PLoS One. 2009 Aug 27;4(8):e6809. doi: 10.1371/journal.pone.0006809 (PMC2728540; doi:10.1371/journal.pone.0006809)
Supplement: Table S3 — Sex-specific odds ratios of future coronary artery disease by quartiles of PON1 activity and by quartiles of PON-1 activity adjusted for PON1-192 genotype. (0.06 MB DOC) [file pone.0006809.s003.doc]

**Table S3: Sex-specific odds ratios of future coronary artery disease by quartiles of PON1 activity and by quartiles of PON-1 activity adjusted for PON1-192 genotype**

| **Men** |  |  |  |  |  |
| --- | --- | --- | --- | --- | --- |
| **PON1 activity, U/l** | **1^st^ quartile** | **2^nd^ quartile** | **3^rd^ quartile** | **4^th^ quartile** | **P** |
|  | **(< 25.9)** | **(25.9 – 43.0)** | **(43.0 – 89.9)** | **(> 89.9)** |  |
| Cases / controls | 223 / 373 | 157 / 345 | 196 / 367 | 149 / 326 |  |
| OR unadjusted | 1.0 | 0.74 (0.57-0.95) | 0.89 (0.70-1.13) | 0.75 (0.58-0.98) | 0.07 |
| OR adjusted for HDL parameters, (1) | 1.0 | 0.87 (0.64-1.19) | 0.90 (0.68-1.20) | 0.81 (0.58-1.12) | 0.2 |
| OR adjusted for (1) + risk factors, (2) | 1.0 | 0.87 (0.60-1.24) | 0.98 (0.70-1.36) | 0.81 (0.55-1.19) | 0.5 |
| **Activity adjusted for 192 genotype, AU/l** | **(< -16.8)** | **(-16.8 to -7.8)** | **(-7.8 to 5.6)** | **(> 5.6)** | **P** |
| Cases / controls | 223 / 394 | 206 / 352 | 147 / 327 | 141 /325 |  |
| OR unadjusted | 1.0 | 1.02 (0.79-1.31) | 0.78 (0.60-1.02) | 0.74 (0.57-0.97) | 0.009 |
| OR adjusted for HDL parameters, (1) | 1.0 | 1.07 (0.82-1.40) | 0.94 (0.70-1.26) | 0.93 (0.70-1.26) | 0.5 |
| OR adjusted for (1) + risk factors, (2) | 1.0 | 1.16 (0.82-1.64) | 1.00 (0.69-1.46) | 0.93 (0.63-1.37) | 0.6 |
| **Woman** |  |  |  |  |  |
| **PON1 activity, U/l** | **1^st^ quartile** | **2^nd^ quartile** | **3^rd^ quartile** | **4^th^ quartile** | **P** |
|  | **(< 25.9)** | **(25.9 – 43.0)** | **(43.0 – 89.9)** | **(> 89.9)** |  |
| Cases / controls | 99 / 149 | 101 / 241 | 88 / 193 | 125 / 243 |  |
| OR unadjusted | 1.0 | 0.61 (0.42-0.87) | 0.67 (0.47-0.96) | 0.76 (0.55-1.06) | 0.04 |
| OR adjusted for HDL parameters, (1) | 1.0 | 0.55 (0.36-0.85) | 0.66 (0.43-1.02) | 0.70 (0.47-1.06) | 0.3 |
| OR adjusted for (1) + risk factors, (2) | 1.0 | 0.52 (0.31-0.88) | 0.57 (0.34-0.97) | 0.66 (0.40-1.10) | 0.3 |
| **Activity adjusted for 192 genotype, AU/l** | **(< -16.8)** | **(-16.8 to -7.8)** | **(-7.8 to 5.6)** | **(> 5.6)** |  |
| Cases / controls | 87 / 152 | 85 / 174 | 102 / 236 | 119 / 241 |  |
| OR unadjusted | 1.0 | 0.82 (0.56-1.20) | 0.72 (0.50-1.03) | 0.84 (0.60-1.19) | 0.3 |
| OR adjusted for HDL parameters, (1) | 1.0 | 0.99 (0.80-1.24) | 0.85 (0.67-1.06) | 0.94 (0.74-1.18) | 0.3 |
| OR adjusted for (1) + risk factors, (2) | 1.0 | 0.64 (0.37-1.12) | 0.42 (0.24-0.74) | 0.61 (0.36-1.03) | 0.1 |

Odds ratios (ORs) for the risk of future CAD events by quartiles of PON1 activity and ORs by quartiles of PON1 activity adjusted for PON1-192 genotype (95% Confidence Interval). (1) Adjustment for HDL parameters (HDL particle number, HDL-cholesterol, HDL size and apoA-I), (2) Adjustment for HDL parameters and risk factors (alcohol use, BMI, CRP, diabetes mellitus, fasting time, LDL cholesterol, myeloperoxidase, smoking, systolic blood pressure, vitamin C, vitamin supplement use, waist circumference, triglycerides). P= p-value for trend = χ^2^ linear trend with 1 degree of freedom. AU indicates arbitrary units. A total of 1138 cases and 2237 controls were included in the analysis from which 1099 cases were matched to two controls and 39 cases were matched to one control. Data on PON1 genotype was missing in 61 cases. Values can be based on a lower number of subjects for some variables.
