# Supplementary material for: Both Paraoxonase-1 Genotype and Activity Do Not Predict the Risk of Future Coronary Artery Disease; the EPIC-Norfolk Prospective Population Study
Source: PLoS One. 2009 Aug 27;4(8):e6809. doi: 10.1371/journal.pone.0006809 (PMC2728540; doi:10.1371/journal.pone.0006809)
Supplement: Table S4 — Backward Stepwise Cox Regression Analysis: HDL-cholesterol with all and excluded variables. (0.08 MB DOC) [file pone.0006809.s004.doc]

**Table S4. Backward Stepwise Cox Regression Analysis: HDL-cholesterol with all and excluded variables**

| **A** | **Variables in the equation** |  |  | **95%** | **CI** |
| --- | --- | --- | --- | --- | --- |
| **Step 1** |  | **P** | **Odds Ratio** | **Lower** | **Upper** |
|  | Smoking | .000 | 0.702 | .602 | .819 |
|  | Waist circumference | .192 | 1.012 | .994 | 1.031 |
|  | Body mass index | .324 | 1.025 | .976 | 1.076 |
|  | Systolic blood pressure | .003 | 1.008 | 1.003 | 1.014 |
|  | Diabetes Mellitus | .000 | .194 | .108 | .348 |
|  | LDL-cholesterol | .155 | 1.133 | .954 | 1.345 |
|  | Vitamin C | .000 | .990 | .985 | .996 |
|  | Vitamin supplements | .452 | 1.079 | .886 | 1.314 |
|  | Alcohol units | .072 | .990 | .978 | 1.001 |
|  | Fasting time | .066 | 1.001 | 1.000 | 1.001 |
|  | HDL-cholesterol | .058 | .726 | .521 | 1.011 |
|  | Apolipoprotein-B | .145 | 1.005 | .998 | 1.011 |
|  | Myeloperoxidase | .018 | 1.000 | 1.000 | 1.000 |
|  | Triglycerides | .101 | 1.263 | .955 | 1.671 |
|  | C-reactive protein | .040 | 1.099 | 1.005 | 1.203 |
|  | PON1-activity | .340 | .999 | .996 | 1.002 |
|  | PON1-192 genotype | .217 | 1.076 | .958 | 1.210 |
|  | PON1-55 genotype | .424 | 1.042 | .942 | 1.151 |
|  | PON1-activity adjusted for PON1-192 genotype | - |  |  |  |
|  | PON1-activity adjusted for PON1-55 genotype | - |  |  |  |
| **Step 10** | Smoking | .000 | .710 | .610 | .826 |
|  | Waist circumference | .000 | 1.020 | 1.010 | 1.031 |
|  | Systolic blood pressure | .002 | 1.009 | 1.003 | 1.014 |
|  | Diabetes Mellitus | .000 | .208 | .116 | .372 |
|  | Vitamin C | .000 | .991 | .985 | .996 |
|  | Alcohol units | .054 | .989 | .978 | 1.000 |
|  | Fasting time | .056 | 1.001 | 1.000 | 1.001 |
|  | HDL-cholesterol | .010 | .671 | .495 | .908 |
|  | Apolipoprotein-B | .000 | 1.009 | 1.006 | 1.013 |
|  | Myeloperoxidase | .017 | 1.000 | 1.000 | 1.000 |
|  | C-reactive protein | .038 | 1.099 | 1.005 | 1.201 |
| **B** | **Variables not in the equation** | **P** |  |  |  |
| **Step 10** | Body mass index | .314 |  |  |  |
|  | LDL-cholesterol | .342 |  |  |  |
|  | Vitamin supplements | .457 |  |  |  |
|  | Triglycerides | .235 |  |  |  |
|  | PON1-activity | .656 |  |  |  |
|  | PON1-192 genotype | .435 |  |  |  |
|  | PON1-55 genotype | .199 |  |  |  |
|  | PON1-activity adjusted for PON1-192 genotype | .245 |  |  |  |
|  | PON1-activity adjusted for PON1-55 genotype | .934 |  |  |  |
