# Supplementary material for: Both Paraoxonase-1 Genotype and Activity Do Not Predict the Risk of Future Coronary Artery Disease; the EPIC-Norfolk Prospective Population Study
Source: PLoS One. 2009 Aug 27;4(8):e6809. doi: 10.1371/journal.pone.0006809 (PMC2728540; doi:10.1371/journal.pone.0006809)
Supplement: Table S5 — Backward Stepwise Cox Regression Analysis: HDL-particles with all and excluded variables. (0.08 MB DOC) [file pone.0006809.s005.doc]

**Table S5.** **Backward Stepwise Cox Regression Analysis: HDL-particles with all and excluded variables**

| **A** | **Variables in the equation** |  |  | **95%** | **CI** |
| --- | --- | --- | --- | --- | --- |
|  |  | **P** | **Odds Ratio** | **Lower** | **Upper** |
| **Step 1** | Smoking | .000 | .677 | .579 | .792 |
|  | Waist circumference | .304 | 1.010 | .991 | 1.029 |
|  | Body mass index | .192 | 1.033 | .984 | 1.086 |
|  | Systolic blood pressure | .001 | 1.010 | 1.004 | 1.015 |
|  | Diabetes Mellitus | .000 | .179 | .097 | .329 |
|  | LDL-cholesterol | .129 | 1.144 | .962 | 1.362 |
|  | Vitamin C | .002 | .992 | .986 | .997 |
|  | Vitamin supplements | .423 | 1.085 | .888 | 1.326 |
|  | Alcohol units | .254 | .993 | .982 | 1.005 |
|  | Fasting time | .047 | 1.001 | 1.000 | 1.001 |
|  | HDL particles | .000 | .951 | .931 | .971 |
|  | Apolipoprotein-B | .250 | 1.004 | .997 | 1.010 |
|  | Myeloperoxidase | .068 | 1.000 | 1.000 | 1.000 |
|  | Triglycerides | .001 | 1.621 | 1.227 | 2.142 |
|  | C-reactive protein | .064 | 1.090 | .995 | 1.194 |
|  | PON1-activity | .495 | .999 | .996 | 1.002 |
|  | PON1-192 genotype | .242 | 1.074 | .953 | 1.211 |
|  | PON1-55 genotype | .223 | 1.065 | .962 | 1.180 |
|  | PON1-activity adjusted for PON1-192 genotype | - |  |  |  |
|  | PON1-activity adjusted for PON1-55 genotype | - |  |  |  |
| **Step 10** | Smoking | .000 | .682 | .585 | .797 |
|  | Body mass index | .000 | 1.056 | 1.025 | 1.087 |
|  | Systolic blood pressure | .001 | 1.010 | 1.004 | 1.015 |
|  | Diabetes Mellitus | .000 | .180 | .098 | .329 |
|  | LDL-cholesterol | .000 | 1.237 | 1.125 | 1.360 |
|  | Vitamin C | .001 | .991 | .986 | .997 |
|  | Fasting time | .047 | 1.001 | 1.000 | 1.001 |
|  | HDL-particles | .000 | .947 | .928 | .966 |
|  | Myeloperoxidase | .079 | 1.000 | 1.000 | 1.000 |
|  | Triglycerides | .000 | 1.802 | 1.423 | 2.281 |
|  | C-reactive protein | .044 | 1.097 | 1.002 | 1.201 |
| **B** | **Variables not in the equation** | **P** |  |  |  |
| **Step 10** | Waist circumference | .414 |  |  |  |
|  | Vitamin supplements | .429 |  |  |  |
|  | Alcohol units | .288 |  |  |  |
|  | Apolipoprotein-B | .312 |  |  |  |
|  | PON1-activity | .654 |  |  |  |
|  | PON1-192 genotype | .396 |  |  |  |
|  | PON1-55 genotype | .144 |  |  |  |
|  | PON1-activity adjusted for PON1-192 genotype | .216 |  |  |  |
|  | PON1-activity adjusted for PON1-55 genotype | .978 |  |  |  |
