# Supplementary material for: Both Paraoxonase-1 Genotype and Activity Do Not Predict the Risk of Future Coronary Artery Disease; the EPIC-Norfolk Prospective Population Study
Source: PLoS One. 2009 Aug 27;4(8):e6809. doi: 10.1371/journal.pone.0006809 (PMC2728540; doi:10.1371/journal.pone.0006809)
Supplement: Table S6 — Backward Stepwise Cox Regression Analysis: apolipoproteinA-I with all and excluded variables. (0.08 MB DOC) [file pone.0006809.s006.doc]

**Table S6.** **Backward Stepwise Cox Regression Analysis: apolipoproteinA-I with all and excluded variables**

| **A** | **Variables in the equation** |  |  | **95%** | **CI** |
| --- | --- | --- | --- | --- | --- |
|  |  | **P** | **Odds Ratio** | **Lower** | **Upper** |
| **Step 1** | Smoking | .000 | .681 | .580 | .801 |
|  | Waist circumference | .138 | 1.015 | .995 | 1.034 |
|  | Body mass index | .405 | 1.022 | .971 | 1.076 |
|  | Systolic blood pressure | .003 | 1.009 | 1.003 | 1.015 |
|  | Diabetes Meliitus | .000 | .235 | .128 | .431 |
|  | LDL-cholesterol | .733 | 1.032 | .860 | 1.240 |
|  | Vitamin C | .005 | .992 | .986 | .998 |
|  | Vitamin supplements | .714 | 1.040 | .844 | 1.281 |
|  | Alcohol units | .185 | .992 | .980 | 1.004 |
|  | Fasting time | .079 | 1.001 | 1.000 | 1.001 |
|  | ApoA1 | .001 | .993 | .989 | .997 |
|  | Apolipoprotein-B | .009 | 1.009 | 1.002 | 1.016 |
|  | Myeloperoxidase | .055 | 1.000 | 1.000 | 1.000 |
|  | Triglycerides | .231 | 1.194 | .893 | 1.597 |
|  | C-reactive protein | .041 | 1.105 | 1.004 | 1.216 |
|  | PON1-activity | .316 | .998 | .995 | 1.002 |
|  | PON1-192 genotype | .296 | 1.068 | .944 | 1.208 |
|  | PON1-55 genotype | .370 | 1.049 | .944 | 1.166 |
|  | PON1-activity adjusted for PON1-192 genotype | - |  |  |  |
|  | PON1-activity adjusted for PON1-55 genotype | - |  |  |  |
| **Step 11** | Smoking | .000 | .699 | .596 | .819 |
|  | Waist circumference | .000 | 1.022 | 1.010 | 1.033 |
|  | Systolic blood pressure | .003 | 1.009 | 1.003 | 1.015 |
|  | Diabetes Mellitus | .000 | .245 | .135 | .447 |
|  | Vitamin C | .002 | .992 | .986 | .997 |
|  | Fasting time | .082 | 1.001 | 1.000 | 1.001 |
|  | ApoA-I | .000 | .992 | .988 | .996 |
|  | Apolipoprotein-B | .000 | 1.011 | 1.008 | 1.015 |
|  | Myeloperoxidase | .050 | 1.000 | 1.000 | 1.000 |
|  | C-reactive protein | .028 | 1.112 | 1.012 | 1.222 |
| **B** | **Variables not in the equation** | **P** |  |  |  |
| **Step 11** | Body mass index | .325 |  |  |  |
|  | LDL-cholesterol | .752 |  |  |  |
|  | Vitamin supplements | .798 |  |  |  |
|  | Alcohol units | .141 |  |  |  |
|  | Triglycerides | .222 |  |  |  |
|  | PON1-activity | .397 |  |  |  |
|  | PON1-192 genotype | .637 |  |  |  |
|  | PON1-55 genotype | .172 |  |  |  |
|  | PON1-activity adjusted for PON1-192 genotype | .159 |  |  |  |
|  | PON1-activity adjusted for PON1-55 genotype | .633 |  |  |  |
