# Supplementary material for: Both Paraoxonase-1 Genotype and Activity Do Not Predict the Risk of Future Coronary Artery Disease; the EPIC-Norfolk Prospective Population Study
Source: PLoS One. 2009 Aug 27;4(8):e6809. doi: 10.1371/journal.pone.0006809 (PMC2728540; doi:10.1371/journal.pone.0006809)
Supplement: Table S7 — Distribution of combined PON1-haplotypes and its effect on HDL-cholesterol, HDL-particles, PON1-acitivity and PON1-activity adjusted for PON1-192 genotype. (0.04 MB DOC) [file pone.0006809.s007.doc]

**Table S7. Distribution of combined PON1-haplotypes and its effect on HDL-cholesterol, HDL-particles, PON1-acitivity and PON1-activity adjusted for PON1-192 genotype**

| **Haplotypes** | **Total, n** | **Controls** | **Cases** | **HDL-cholesterol** | **HDL-particles** | **PON1-activity** | **Activity adjusted for genotype** |
| --- | --- | --- | --- | --- | --- | --- | --- |
| **LQ/MQ** | 761 | 498 | 263 | 1.31±0.4 | 33.5±5.5 | 24.6±7.4 | -13±7 |
| **MQ/LR** | 629 | 417 | 212 | 1.34±0.4 | 34.1±5.7 | 85±28 | -5±28 |
| **LQ/LR** | 585 | 393 | 192 | 1.35±0.4 | 33.7±6.1 | 90±27 | 1±27 |
| **LQ/LQ** | 423 | 293 | 130 | 1.33±0.4 | 33.9±5.4 | 33±9 | -3±9 |
| **MQ/MQ** | 384 | 248 | 136 | 1.30±0.5 | 36.1±3.8 | 21±7 | -15±7 |
| **LR/LR** | 254 | 163 | 91 | 1.35±0.4 | 34.2±5.5 | 152±45 | 89±45 |
| **MQ/MR** | 13 | 10 | 3 | 1.30±0.3 | 35.3±5.7 | 76±29 | -14±29 |
| **LR/MR** | 8 | 7 | 1 | 1.24±0.5 | 36.3±4.9 | 166±22 | 103±22 |

Data are presented as mean ± standard deviation.

Data are presented as mean (+SD) standard deviation. In 318 samples we could not determine PON1-haplotypes (missing data).
